# Acute Hepatitis B and Acute HIV Coinfection in an Adult Patient: A Rare Case Report

**DOI:** 10.1155/2010/820506

**Published:** 2010-11-07

**Authors:** Raghav Bansal, Maurice Policar, Chinar Mehta

**Affiliations:** ^1^Department of Medicine, Mount Sinai Elmhurst Hospital Center, 7901 Broadway, Elmhurst, NY 11373, USA; ^2^Division of Infectious Diseases, Mount Sinai Elmhurst Hospital Center, 7901 Broadway, Elmhurst, NY 11373, USA; ^3^Department of Gastroenterology, Mount Sinai Elmhurst Hospital Center, 7901 Broadway, Elmhurst, NY 11373, USA

## Abstract

Acute HIV and acute hepatitis B coinfection is extremely rare. A 23-year-old homosexual man was admitted to our hospital with 5-day history of fever, malaise, and back pain with initial laboratory values showing severe transaminitis. The clinical picture was initially suggestive of acute viral hepatitis, which on further testing revealed acute hepatitis B and acute HIV coinfection. Although the patient was asymptomatic, a decision was made to start antiretroviral therapy. At 2-month followup, liver function tests were normal with undetectable viral loads. The early treatment of acute HIV/HBV coinfections likely contributed to eventual seroconversion with immunity to HBV in a severely immunocompromised host. To the best of our knowledge, this is the first case report of acute Hepatitis B and acute HIV coinfection and its management. In conclusion, early treatment of acute hepatitis B in immunocompromised patients may be beneficial.

## 1. Introduction

Coinfection with HBV is common in HIV-infected individuals because of their shared routes of transmission. Chronic hepatitis B virus (HBV) infection affects approximately 7% to 15% of HIV-infected persons worldwide [1]. The incidence of complications arising from HBV-associated liver disease among coinfected patients represents a significant challenge for both patients and clinicians. Management of coinfection has always been challenging because of interaction between two viruses, deciding when to initiate the therapy, dual action of some drugs, and morbidity and mortality associated with these diseases. We report a case of simultaneous acute HBV and acute HIV coinfection and its management.

## 2. Case Report

A 23-year-old homosexual male without past medical history presented with a 5-day history of fever, malaise, nausea, and back pain. Social history was remarkable for multiple sexual partners, inconsistent condom use, smoking 4-5 cigarettes/day, and drinking 6 beers/weekend. Physical examination was normal. Vital signs were normal except for a temperature of 101°F. Laboratory values at admission are shown (Table 1). By hospital day no. 5, liver function tests (LFTs) continued to increase. After consulting with a gastroenterologist and infectious disease specialist, a decision was made to treat both infections with tenofovir/emtricitabine and ritonavir/lopinavir although the patient was asymptomatic. 

By hospital day no. 7, when antiviral medications were dispensed to the patient, LFTs had begun to decrease.

Laboratory values at 2 months are shown in Table 1.

At 17-month followup, pt remained stable on antiretroviral therapy.

## 3. Discussion

The prevalence of HIV/HBV coinfection varies. The geographical areas with the highest prevalence are in sub-Saharan Africa and Asia, and the type of risk behavior with the highest prevalence is male homosexuality [2]. There appears to be a correlation between higher CD4+ T-cell counts and clearance of HBV viremia during acute HBV infection [3]. HIV-infected individuals have an increased incidence of nonicteric illness, higher levels of HBV DNA, an increased rate of developing chronic hepatitis (23%), and subsequent reactivation of hepatitis B [4]. Rates of seroconversion are lower in HIV-infected individuals. In one study of HBV carriers, HbeAg loss over a 5-year period was 12% in coinfected patients, compared with 49% in those with HBV alone [5]. Most studies have shown no significant influence of HBV infection on the progression of HIV disease [4, 5]. 

There is little evidence to support treatment of acute hepatitis B infection except in severe, protracted, or fulminant cases [6]. Lamivudine has been administered successfully to patients who develop de novo Hepatitis B after liver transplantation [7, 8]. Treatment of acute hepatitis B infection alone in patients with underlying HIV infection is further complicated by potential for emergence of HIV resistance mutations because of dual action of some drugs, thereby limiting further treatment options.

The diagnosis of Acute HIV infection is established by documenting a high viral load in a patient with typical clinical features and a negative or indeterminate HIV Antibody test. In our patient, the Rapid HIV test used was the FDA-approved OraQuick ADVANCE Rapid HIV-1/2 Antibody Test from OraSure Technologies, using whole blood. It is extremely sensitive and does not require confirmation for a negative test. The HIV viral load was determined using the Roche Ambliprep/Cobas (R) Taqman (R) HIV-1 test kit. 

The need for treatment of acute HIV infection is still debatable. An early report showed that persons with symptomatic HIV infection had a more rapidly progressive course, and the authors suggested treatment for those patients [9]. The benefits of initiating early treatment of acute HIV infection are unclear, and the long-term issues of toxicities, resistance, quality of life, and cost remain. An additional concern in the setting of HBV infection in HIV/HBV coinfection would include worsening transaminitis due to immune reconstitution or because of antiretroviral-associated hepatotoxicity. 

There is no specific guidance on how to manage patients that may have simultaneous acute coinfection with HIV and Hepatitis B, such as our patient [10]. For chronically coinfected patients, guidelines recommend initiation of antiretroviral therapy in those initiating antihepatitis B therapy, in order to avoid developing HIV resistance to agents that treat both viral infections. For patients with chronic HIV infection who develop acute HBV infection, there are no specific recommendations other than to avoid initiating antiretroviral therapy (other than lamivudine) in those with fulminant liver disease [11]. 

After much consideration and review of available evidence, we decided to manage our patient with antiretroviral therapy that treats HBV. We believe that there is sufficient evidence to support treatment of acute HIV with antiretroviral therapy. Although our patient did not have evidence of fulminant hepatits, the continued increase in transaminase levels was concerning. Treatment in our patient may have contributed to eventual seroconversion with immunity to HBV. It is clear that patients with chronic HIV infection are less likely to seroconvert. The rate of clearance of HBV in persons with acute HIV infection is unknown but likely to be even less than those with chronic HIV. 

A previous report of concurrent HBV and HIV infections described a case of acute HBV infection with a negative HIV test, followed by a positive HIV test several months later [12]. To the best of our knowledge, this is the first case report of simultaneous acute HBV and acute HIV coinfection and management.

## Figures and Tables

**Table 1:** Laboratory values.

	Day 1	Day 5	Day 7	2 months	17 months
WBC (K/mcl)	1.7	2.6	3.8	5.7	6.2
Platelets (K/mcl)	101	101	203	208	165
Sodium (mEq/L)	131	135	134	133	135
AST (U/L)	2920	3801	1889	18	19
ALT (U/L)	3772	4951	4092	33	18
Alkaline phosphatase	82	111	119	53	64
Gamma-glutamyl transpeptidase	343		423	45	23
Albumin(g/dL)	3.4	3.3	3.7	4.0	4.3
Total bilirubin (m/dL)	0.9	2.5	4.2	1.2	1.4
INR	1.35	0.98	1.06		
PT (seconds)	15.6	11.3	12.2		
HBsAg	+			−	
HBeAg	+			−	
HBcAb IgM	+			+	
Total HBcAb	+			+	
HBeAb	−			+	
HBsAb	−			+	
Rapid HIV Ab	−			+	
CD4 count (/cumm)	120			469	840
HIV RNA PCR (copies/mL)	>10,000,000			651	<48
HBV DNA PCR (copies/mL)	22,613,715			<160	
HAV Ab IgM	−				
HCV Ab	−				
HCV RNA (IU/mL)	<50				
